# High vector diversity and malaria transmission dynamics in five sentinel sites in Cameroon

**DOI:** 10.1186/s12936-023-04552-z

**Published:** 2023-04-13

**Authors:** Etienne Fondjo, Jean-Claude Toto, Magellan Tchouakui, Wolfgang Ekoko Eyisap, Salomon Patchoke, Benjamin Menze, Boris Njeambosay, Francis Zeukeug, Raymond Tabue Ngomdjum, Elysée Mandeng, Emmanuel Elanga-Ndille, Edmond Kopya, Jerome Achille Binyang, Cyrille Ndo, Billy Tene-Fossog, Armel Tedjou, Elysée Nchoutpouen, Frederic Tchouine, Dorothy Achu, Kelley Ambrose, Judith Hedje, Celestin Kouambeng, Jenny Carlson, Sarah Zohdy, Joseph Chabi

**Affiliations:** 1 U.S. President’s Malaria Initiative (PMI) VectorLink Project, Abt Associates, Yaoundé, Cameroon; 2 Central African Organization for Endemic Disease Control (OCEAC), Yaoundé, Cameroon; 3 Centre for Research in Infectious Diseases (CRID), Yaoundé, Cameroon; 4 University of Bamenda, Bamenda, Cameroon; 5 The Biotechnology Center (BTC), University of Yaoundé 1, Yaoundé, Cameroon; 6 National Malaria Control Programme, Yaoundé, Cameroon; 7 U.S. President’s Malaria Initiative VectorLink Project, Abt Associates, Rockville, MD USA; 8 U.S. President’s Malaria Initiative, U.S. Centers for Disease Control and Prevention (CDC), Yaoundé, Cameroon; 9 U.S. President’s Malaria Initiative U.S. Agency for International Development (USAID), Yaoundé, Cameroon; 10 U.S. President’s Malaria Initiative, USAID, Washington, DC USA; 11 U.S. President’s Malaria Initiative, U.S. Centers for Disease Control and Prevention (CDC), Atlanta, GA USA

**Keywords:** Malaria transmission, Vector diversity, Vectorial capacity, Cameroon

## Abstract

**Background:**

Malaria remains one of the main causes of morbidity and mortality in Cameroon. To inform vector control intervention decision making, malaria vector surveillance was conducted monthly from October 2018 to September 2020 in five selected sentinel sites (Gounougou and Simatou in the North, and Bonabéri, Mangoum and Nyabessang in the South).

**Methods:**

Human landing catches (HLCs), U.S. Centers for Disease Control and Prevention (CDC) light traps, and pyrethrum spray catches (PSCs) were used to assess vector density, species composition, human biting rate (HBR), endophagic index, indoor resting density (IRD), parity, sporozoite infection rates, entomological inoculation rate (EIR), and *Anopheles* vectorial capacity.

**Results:**

A total of 139,322 *Anopheles* mosquitoes from 18 species (or 21 including identified sub-species) were collected across all sites. Out of the 18 species, 12 were malaria vectors including *Anopheles gambiae sensu lato* (s.l.), *Anopheles funestus* s.l.., *Anopheles nili*, *Anopheles moucheti*, *Anopheles paludis*, *Anopheles demeilloni*, *Anopheles. pharoensis*, *Anopheles ziemanni*, *Anopheles multicinctus*, *Anopheles tenebrosus*, *Anopheles rufipes*, and *Anopheles marshallii*. *Anopheles gambiae* s.l. remains the major malaria vector (71% of the total *Anopheles*) collected, though *An. moucheti* and *An. paludis* had the highest sporozoite rates in Nyabessang. The mean indoor HBR of *Anopheles* ranged from 11.0 bites/human/night (b/h/n) in Bonabéri to 104.0 b/h/n in Simatou, while outdoors, it varied from 24.2 b/h/n in Mangoum to 98.7 b/h/n in Simatou. *Anopheles gambiae s.l*. and *An. moucheti* were actively biting until at least 8:00 a.m. The mean *Anopheles* IRD was 17.1 females/room, and the parity rate was 68.9%. The mean EIRs for each site were 55.4 infective bites/human/month (ib/h/m) in Gounougou, 99.0 ib/h/m in Simatou, 51.2 ib/h/m in Mangoum, 24.4 ib/h/m in Nyabessang, and 18.1 ib/h/m in Bonabéri. *Anopheles gambiae* s.l. was confirmed as the main malaria vector with the highest vectorial capacity in all sites based on sporozoite rate, except in Nyabessang.

**Conclusion:**

These findings highlight the high malaria transmission occurring in Cameroon and will support the National Malaria Control Program to design evidence-based malaria vector control strategies, and deployment of effective and integrated vector control interventions to reduce malaria transmission and burden in Cameroon, where several *Anopheles* species could potentially maintain year-round transmission.

**Supplementary Information:**

The online version contains supplementary material available at 10.1186/s12936-023-04552-z.

## Background

Malaria remains a leading public health concern in Cameroon, accounting for 29.1% of health facility consultations and 17.2% of deaths in 2020 [1, 2]. Children under five years of age and pregnant women are disproportionately vulnerable. In 2020, hospital morbidity due to malaria was 40.1% among children under five years and 22.5% for pregnant women [1, 2]. In the last two decades, efforts and progress have been made worldwide to control the disease by implementing several vector control measures in addition to therapeutic care. The use of insecticide-treated nets (ITNs) has contributed to the drastic reduction of the disease burden [3, 4]. Nonetheless, sub-Saharan Africa is still at risk and has the most malaria cases and deaths worldwide. According to the 2021 World Malaria Report, there were an estimated 241 million malaria cases recorded in 2020 globally—an increase from 227 million cases in 2019—with the majority of the increased cases reported from countries in the WHO African Region [3, 4].

In Cameroon, the National Malaria Control Programme (NMCP) and its partners have implemented a three-pronged malaria response, including: (i) free distribution of ITNs through mass campaigns and during antenatal consultations for pregnant women, (ii) seasonal malaria chemoprevention for children aged 3 to 59 months, specifically in the North and Far North regions, and (iii) free treatment of uncomplicated and severe malaria for children under five and subsidized case management of malaria for the general population by supporting any malaria case diagnosis and treatment. The country started implementing ITN mass distribution in 2011 and has conducted three mass campaigns (2011, 2015, and 2019) using pyrethroid-only ITNs in all regions. However, the effectiveness of these control measures is being threatened by factors such as the resistance of vectors to the insecticides used in different ITNs [5, 6], the change in vector behaviours, human population behaviour and movement throughout the night [7, 8, 9, 10], and/or the resistance of the *Plasmodium falciparum* parasite to anti-malarial drugs [11, 12]. As in most sub-Saharan African countries, pyrethroid resistance involving different target sites and metabolic resistance within the main vector *Anopheles gambiae sensu lato* (*s.l*.) population is widespread in Cameroon [13, 14, 15, 16]. Furthermore, several potential malaria vectors have emerged as a new challenge for vector control because the existing interventions target specific indoor feeding and resting behaviours of the primary vector species. Cameroon is at particular risk given it hosts several species of *Anopheles* mosquitoes that have been found to carry malaria parasites [12].

The complex vector-parasite ecology in Cameroon requires that malaria control efforts consider all vector species rather than target a single malaria vector. Continuous evaluation of vector bionomics in a changing landscape is required to improve the vector control strategy. While several entomological studies have been conducted in the country to describe malaria transmission parameters, these are often conducted within a short time frame or in a limited number of sites [12, 17-19]. Based on this context, and to provide recent and extensive entomological data to the NMCP, the U.S. President’s Malaria Initiative (PMI) VectorLink project conducted vector surveillance from 2018 to 2020 in five sentinel sites representing four ecological zones in five of the 10 regions of the country. Vector bionomics and malaria transmission parameters were assessed to support the country’s vector control strategy, including the selection and deployment of appropriate evidence-based vector control tools.

## Methods

### Study sites

Cameroon is subdivided into 10 health regions (Southern Region, East, Centre, Littoral, South-West, West, North-West, Adamaoua, North, and Far North Region). Malaria endemicity in the country varies by region, with the highest incidence in the East Region with 180 confirmed cases per thousand inhabitants in 2021 to the lowest in the Far North Region (< 50 cases per 1000). However, the highest morbidity was recorded in the North with more than 40 deaths due to malaria in 2021; the incidence is also high in this region [20]. Two sites (Gounougou and Simatou) were selected in the northern part of the country and three (Mangoum, Nyabessang, and Bonabéri) in the southern part. Gounougou (13.55°E; 9.07°N) is a rice cultivation area located in the dry savannah zone of the North. It has a rainy season of about six months (May to October) and is in one of the high malaria endemicity regions and where the highest morbidity occurred. Simatou (15°E; 10.34°N), situated in the Sahelian zone in the Far North, is also a rice cultivation area, with a short rainy season occurring from July to October and a moderate malaria incidence (119.0 cases per 1000).

**Fig. 1:**
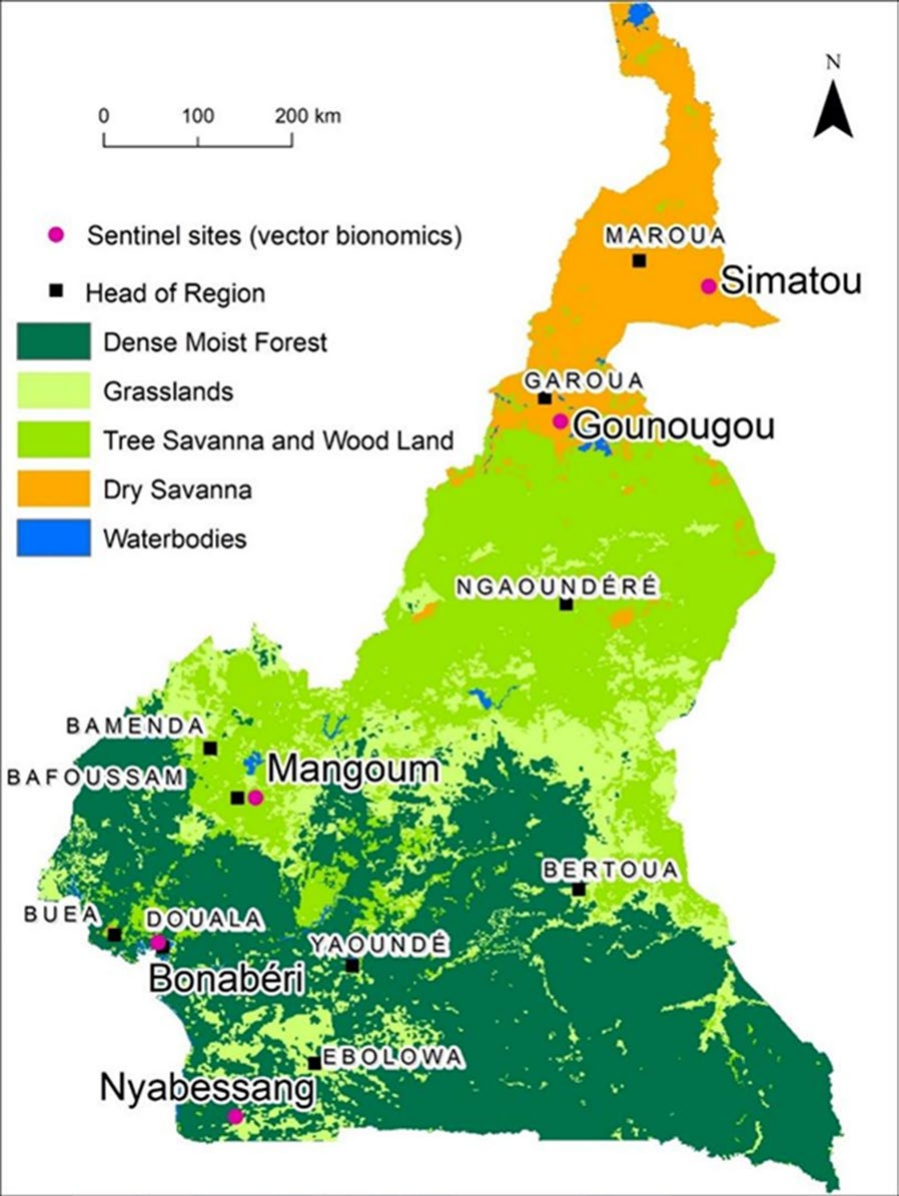
Map of Cameroon showing the geo-location of the five vector surveillance sentinel sites and vegetation across the country

Mangoum (10.58°E; 5.47°N) is in the wet savanna zone in the West region of the country where malaria endemicity is relatively low (96.0 cases per 1000). Nyabessang (10.39°E; 2.4°N), a rural area located in the forest of the South region with high rainfall, is surrounded by many rivers and dams and has low malaria endemicity (94.3 cases per 1000). The final site, Bonabéri (9.65°E; 4.08°N) is an urban area located in the coastal zone of the Littoral region with a low endemicity (92.2 cases per 1000) (Fig. 1).

### Vector bionomics monitoring

The study was conducted in the five sentinel sites from October 2018 to September 2020. Monthly entomological data collections were carried out in Gounougou and Simatou from October 2018 through September 2020 (except for November 2019 through March 2020 when collections were conducted every other month at both sites). Thus, a total of 19 collection-months were completed in these two sites over the survey period. In the southern sites, collections were done every other month in Mangoum and Nyabessang from October 2018 through February 2020 and from December 2018 through February 2020 in Bonabéri. Thereafter, the collections were conducted monthly from June to September 2020 at these three sites. A total of 13 months of field collections were completed in Mangoum and Nyabessang and 12 months in Bonabéri. No collections were conducted between April and May 2020 due to the COVID-19 pandemic. Collections were adjusted to every other month per activity programming including expansion of insecticide resistance monitoring sites across the country. For all collection methods, the same houses were used monthly and throughout the collection period.

Adult mosquitoes were collected in each of the sites using three collection methods: human landing catches (HLCs) which target human host seeking biting vectors, U.S. Centers for Disease Control and Prevention (CDC) light traps which target a diversity of host-seeking species, and pyrethrum spray catches (PSCs) which target indoor resting vectors. Each method enabled the estimation of different parameters which characterized the vector behaviour and malaria transmission within the local populations.

HLCs were conducted in three randomly selected houses per site that were maintained throughout the collection period. Adult mosquito collections were done from 6:00 p.m. to 8:00 a.m. for two consecutive nights per collection month. Four collectors were assigned to each house (two collectors indoors and two collectors outdoors). The collectors were monitored for malaria symptoms before and after collection, and any malaria cases were given treatment. For efficiency and accuracy, two teams of 12 collectors each worked in shifts (from 6:00 p.m. to midnight and from midnight to 8:00 a.m.) and rotated each day. Additionally, the collectors rotated positions every hour throughout the night to account for variation in attractiveness among collectors. The collectors used hemolysis tubes to catch mosquitoes landing on their lower exposed limbs. Mosquitoes were collected hourly and put in separate bags. After each night of collection, mosquitoes were identified morphologically using taxonomic identification keys [21-23]. For each collection month and site, a subsample of randomly selected vectors underwent ovary dissection for parity rate determination [24].

CDC light traps were set indoors and outdoors from 6:00 p.m. to 6:00 a.m. in four houses for two consecutive nights per collection month. The indoor traps were baited and suspended nearby a bed with a mosquito net where household members slept. Outdoor traps were hung on a tree with no bait. All traps were suspended 1.5 m above the ground.

PSCs were carried out during two consecutive days per month in 20 houses (10 houses per day) between 6:00 a.m. and 8:00 a.m. A room in which inhabitants spent the night was selected in each house. A white sheet was placed in the room covering the floor and bed. A pyrethroid insecticide spray containing piperonyl butoxide (PBO) synergist was used to spray the room and to collect all indoor resting mosquitoes. When the house had open eaves, these were sprayed first from outside before spraying indoors to prevent the mosquitoes from escaping. After about 10 min post-spraying, the sheets were gently brought outdoors and the mosquitoes on the sheets were collected using forceps and preserved in petri dishes for morphological identification. The abdominal status of the collected vectors was determined, and the number and percentage of blood-fed mosquitoes were recorded during morphological identification [21].

### **Parity** a**ssessment**

To determine the parity rate of *Anopheles* species collected, approximately 20% of unfed, female *Anopheles* collected using HLCs were randomly selected each month for ovary dissection, following the methods described by Detinova 1962, by observing the degree of coiling by the ovarian tracheoles [24]. All *Anopheles* and the carcasses of the dissected *Anopheles* were individually stored in labeled Eppendorf tubes containing silica gel for further molecular analysis.

### Molecular characterization

A random subsample of about 100 field preserved mosquitoes that were morphologically identified as either *An. gambiae s.l*. and *Anopheles funestus s.l*. (when collected) were selected per month, per collection method, and per site, and used for molecular species identification using polymerase chain reaction (PCR) methods to discriminate between sibling *Anopheles* species using wings and legs. Additionally, about 400 mosquitoes were randomly selected from each of the *Anopheles* species collected to detect sporozoite infections using the head and thorax using indirect enzyme-linked immunosorbent assay (ELISA), and 100 abdomen of PSC-collected *An. gambiae s.l.* were sampled per month to determine blood meal sources using direct ELISA.

### Genomic DNA extraction and species identification

Whole genomic DNA (gDNA) was extracted from each mosquito sample following the LIVAK method [25] and stored at − 20 ºC. A NanoDrop^™^ spectrophotometer (Thermo-Scientific, Wilmington, USA) was used to determine the concentration and purity of the extracted DNA.

Members of *An. gambiae* complex (*An. gambiae sensu stricto* (*s.s*.), *Anopheles coluzzii*, and *Anopheles arabiensis)* were discriminated using the Short Interspersed Nuclear Element (SINE) PCR protocol of Santolamazza et al. [26]. In the coastal sites such as Bonabéri and Nyabessang, where other species, such as *Anopheles melas* are present, the PCR-RFLP protocol described by Fanello et al. [27] was used to discriminate the *An. gambiae* species complex. *Anopheles funestus* group subspecies were characterized using a cocktail PCR according to Koekemoer et al. [28] with the addition of the *Anopheles rivulorum-like* primers. Genomic DNA from 100 randomly selected mosquitoes were processed per month and per site. PCR products were run via electrophoresis through a 1.5% agarose gel with Midori Green® (Gene flow, UK) and visualized under ultraviolet light.

### Circumsporozoite infection detection

Determination of sporozoite infection rates and blood meal analysis of adult *Anopheles* mosquitoes collected using HLCs and PSCs were conducted using circumsporozoite ELISA (csELISA) following the method described by Burkot et al. [29] and modified by Wirtz et al. [30] for sporozoite detection in the head and thorax of mosquitoes. This method uses a monoclonal antibody that recognizes a repetitive epitope on the circumsporozoite protein of *P. falciparum*. *Plasmodium falciparum* sporozoite ELISA reagent kits (MRA-890) were obtained from BEI Resources (NIAID, NIH, USA). Lyophilized *P. falciparum* monoclonal antibody was reconstituted prior to utilization using a glycerol-water solution to achieve a final concentration of 0.5 mg/ml. Similarly, all reagents including phenol red, 1X Phosphate Buffered Saline (PBS), Blocking Buffer (BB), Grinding Buffer, and 1X PBS-Tween wash solution were prepared before starting the manipulation and according to the manufacturer guidance (MR4-890 kit). Diluted *P. falciparum* sporozoite recombinant proteins supplied by CDC (Atlanta, USA) were used as positive controls, while ground male mosquitoes were used as negative controls. Determination of positive samples was done after reading optical densities (OD) at 405 nm on an ELISA plate reader (Biotek Elx800, Swindon, UK). Positive samples were determined by OD readings two-fold greater than the negative controls and were tested a second time for validation.

### Identification of blood meal source

The source of the blood meal contained in the abdomen of resting mosquitoes collected by PSCs was determined using a direct ELISA technique described by Beier et al. [31]. This technique allows the identification of human, cow, pig, chicken, goat, horse, and dog blood. Peroxidase conjugated antibodies and animal heterologous serum were obtained from Sigma (St. Louis, USA). After manipulation, absorbance at 414 nm was determined using an ELISA plate reader. Samples were considered positive if the absorbance values exceeded the mean plus three times the standard deviation of four negative controls represented by unfed mosquitoes.

### Vectorial capacity of *Anopheles* species

The vectorial capacity represents the ability of a population of vectors to transmit *Plasmodium* spp. in terms of the potential number of secondary inoculations originating per day from an infective person. The vectorial capacity is dependent upon a series of biological characteristics such as population density, blood meal preference, and the probability of the vector to survive per day. The MacDonald formula was used to estimate the vectorial capacity of each *Anopheles* species found with *P. falciparum* parasite across all sentinel sites assuming that an infectious person will be subject to m mosquito bites (assuming everyone is equally attractive) and will receive ma bites each day corresponding to the HBR. For those mosquitoes to become infectious they must survive the extrinsic incubation period (with probability p^n^). The adult mosquitoes (on average) live for 1/(− ln (p)) days biting, and potentially infecting, humans at a rate of “a” per day [32]. The equation combines these quantities to give the total potential infectious bite index arising from one infected person for one day, as described below:

$VC=\frac{{\left(m{a}^{2}\right)p}^{n}}{- ln\left(p\right)}$Where a = the ratio of mosquitoes feeding on human, m = ma (man aggressivity) represented by the HBR of the vector, the parasite’s extrinsic incubation period (EIP, n days) which we considered as 12 days, and p = the mosquito’s survival through one day calculated using the parity rate.

### Data management and statistical analysis

All entomological data was regularly entered in Epi-Info Version 3.5.4 by a database manager to facilitate analysis. The proportion of each identified mosquito species was calculated as a percentage of each species out of the total *Anopheles* collected. The mean IRD of each *Anopheles* species collected using PSCs was calculated monthly by dividing the total number of mosquitoes collected by the total number of houses visited. The sporozoite infection rate, measured as the proportion of mosquitoes found with circumsporozoite antigen by ELISA, was calculated by dividing the number of positive mosquitoes by the total number of mosquitoes tested per month. The mean sporozoite rate represents the average of all monthly infection rates per site and per species tested. The mean parity rate was determined by dividing the number of parous females by the total number dissected and averaged over the collection period for the total mean parity rate per site.

The EIR was calculated as the product of the HBR and circumsporozoite antigen rate as determined by ELISA per month, while the mean EIR over the study period represents the average of all monthly EIRs per site and per species. The indoor and outdoor EIRs were compared using the Mann-Whitney test of XLSTAT software for comparing the mean of two series of numbers at 5% significance level. The human blood index (HBI) was calculated as the proportion of mosquitoes found to contain human IgG by ELISA out of the total mosquitoes tested.

## Results

### *Anopheles* mosquito species composition

Overall, 139,326 *Anopheles* mosquitoes representing 18 distinct species were collected in the five sentinel sites using the three methods (HLC, CDC light trap, and PSC). A total of 83,540 *Anopheles* mosquitoes (60.0%) were collected using HLCs; 29,846 (21.4%) were collected with CDC light trap; and 25,940 (18.6%) were collected by PSC. *Anopheles gambiae s.l*. (98,867; 71.0%) was the predominant species and was collected in all sites using the three methods. *Anopheles moucheti* and *Anopheles nili* were only found in Nyabessang (Table 1 and Additional file 1: data 1).

**Table 1:** **Species composition of** ***Anopheles*** mosquitoes collected* by all methods across five sites in Cameroon

Species	Gounougou	Simatou	Mangoum	Nyabessang	Bonabéri	Total collected	Percentage per species collected (%)
*An. gambiae s.l*.	29,514	56,404	6432	1457	5054	98,861	70.96
*An. funestus s.l.*	2623	423	18	10	0	3074	2.21
*An. pharoensis*	341	11,850	0	0	0	12,191	8.75
*An. ziemanni*	673	9429	113	198	1	10,414	7.47
*An. demeilloni*	0	4380	0	0	0	4380	3.14
*An. paludis*	0	1	2	4285	0	4288	3.08
*An. moucheti*	0	0	0	3,546	0	3,546	2.55
*An. rufipes*	339	1126	0	0	0	1465	1.05
*An. nili*	0	0	0	658	0	658	0.47
*An. multicinctus*	226	0	0	0	0	226	0.16
*An. marshallii*	0	0	0	180	0	180	0.13
*An. hancocki*	0	12	0	0	0	12	0.01
*An. tenebrosus*	9	0	0	0	0	9	0.01
*An. welcomei*	0	8	0	0	0	8	0.01
*An. smithii*	5	0	0	0	0	5	0.00
*An. coustani*	1	3	0	0	0	4	0.00
*An. cinereus*	3	0	0	0	0	3	0.00
*An. christyi*	2	0	0	0	0	2	0.00
Total	**33,736**	**83,636**	**6565**	**10,330**	**5055**	**139,326**	**100.0**

*Mosquito collection methods include: HLC: human landing catch; CDC LT: U.S. Centers for Disease Control and Prevention light trap; and PSC: pyrethrum spray catch

A total of 5598 *An. gambiae s.l*. mosquitoes from all five sites were DNA extracted for species identification. Of these, 283 (5.1%) did not amplify, while 5315 *An. gambiae s.l*. (1513 from Simatou, 1,538 from Gounougou, 962 from Mangoum, 575 from Nyabessang, and 727 from Bonabéri) and 596 *An. funestus s.l*. (217 from Simatou, 368 from Gounougou, 9 from Mangoum, and 2 from Nyabessang) were successfully tested by PCR for molecular identification of the sub-species of each complex (Table 2 and Additional file 1: data 1). Three species from the *An. gambiae* complex were identified in Simatou and Gounougou including *An. gambiae s.s*. (1.1% in Simatou, 2.4% in Gounougou), *An. coluzzii* (90.0% in Simatou, 84.2% in Gounougou), and *An. arabiensis* (8.7% in Simatou and 13.4% in Gounougou). Hybrids of *An. gambiae/An. coluzzii* (0.2%) were also recorded in Simatou.

**Table 2:** Species composition of *Anopheles gambiae* complex and *An. funestus* group collected across five sites in Cameroon

Sites	*An. gambiae s.l*.				Total *An. gambiae s.l.*	*An. funestus s.l*.		Total *An. funestus s.l.*
	***An. gambiae s.s*.** **(%)**	***An. coluzzii* (%)**	***An. arabiensis* (%)**	***An. coluzzii/An. gambiae *(%)**		***An. funestus s.s*. (%)**	***An. leesoni* (%)**	
Simatou	17 (1.1%)	1,362 (90.0%)	131 (8.7%)	3 (0.2%)	**1513**	129 (59.4%)	88 (40.6%)	**217**
Gounougou	37 (2.4%)	1,295 (84.2%)	206 (13.4%)	0 (0.0%)	**1538**	330 (89.7%)	38 (10.3%)	**368**
Mangoum	951 (98.9%)	6 (0.6%)	1 (0.1%)	4 (0.4%)	**962**	9 (100.0%)	0	**9**
Nyabessang	538 (93.6%)	30 (5.2%)	0 (0.0%)	7 (1.2%)	**575**	0 (0.0%)	2 (100.0%)	**2**
Bonabéri	10 (1.4%)	717 (98.6%)	0 (0.0%)	0 (0.0%)	**727**	0 (0.0%)	0 (0.0%)	**0**
Total	**1553 (29.2%)**	**3410 (64.2%)**	**338 (6.4%)**	**14 (0.3%)**	**5315**	**468 (78.5%)**	**128 (21.5%)**	**596**

In the three southern sites, two species of the *An. gambiae* complex were recorded, including *An. gambiae s.s*. (98.9% in Mangoum, 93.5% in Nyabessang, and 1.4% in Bonabéri) and *An. coluzzii* (0.6% in Mangoum, 5.2% in Nyabessang, and 98.6% in Bonabéri). A small proportion of hybrids of both species was also recorded in Mangoum (0.4%) and Nyabessang (1.2%).

For *An. funestus s.l*., two species of the group were found in Simatou and Gounougou: *An. funestus s.s*. (59.4% and 89.7%, respectively) and *Anopheles leesoni* (40.5% and 10.3%, respectively). Prior to molecular identification, a second morphological identification was conducted by the laboratory team to ensure that the tested samples were *An. funestus s.l*. to avoid misidentification of the *An. leesoni* that can occur [33] and all detected *An. leesoni* were re-run using a mix with *An. gambiae* primers to confirm the species.

### Malaria transmission parameter’s estimates

#### Malaria vectors and human biting rates

**Fig. 2:**
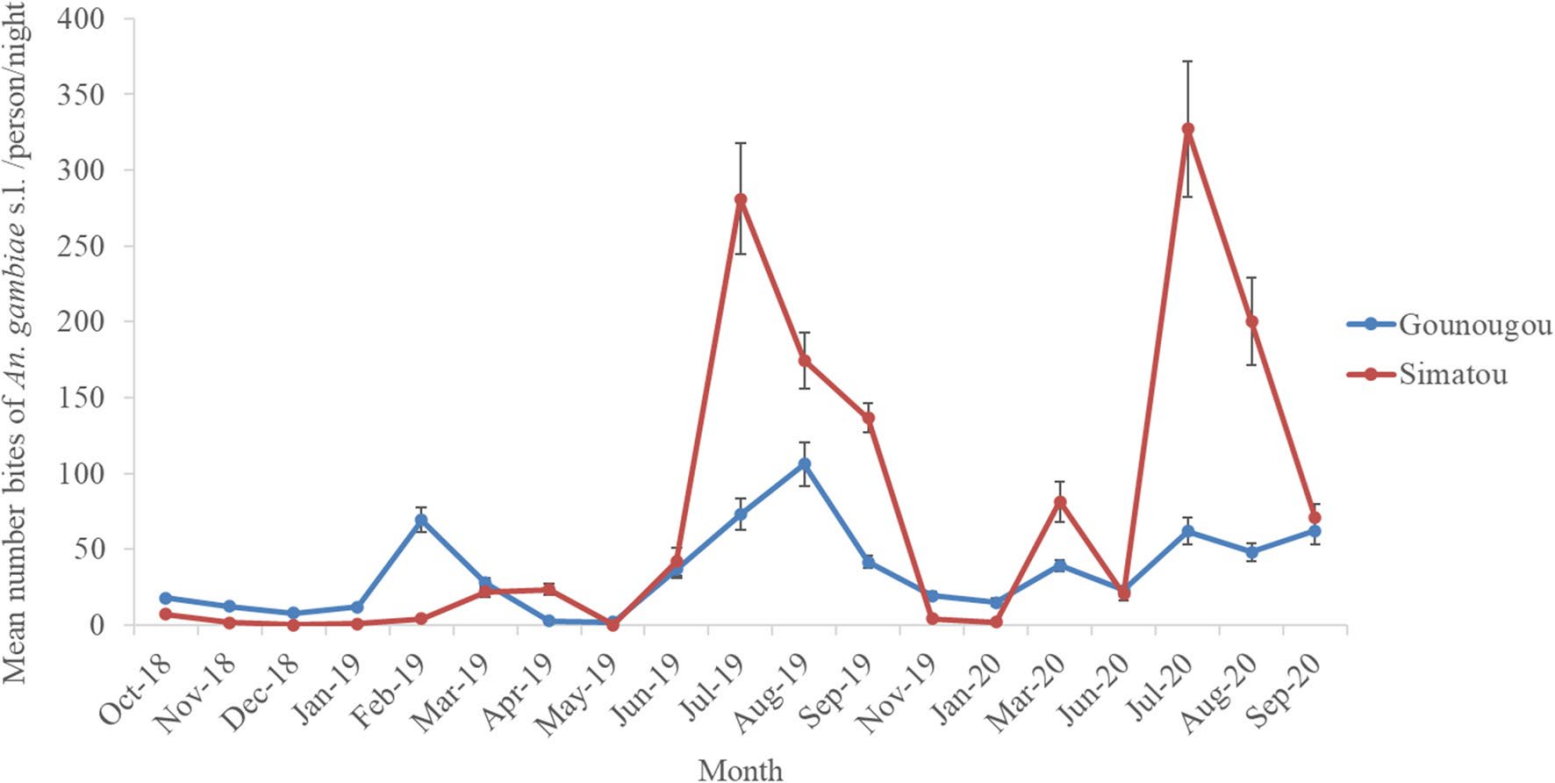
Mean monthly human biting rates of Gounougou and Simatou (Northern sites) over the collection period (October 2018-September 2020)

The HLC method was the most productive collection method at all sites. Of the 83,540 *Anopheles* mosquitoes collected, *An. gambiae s.l*. represented the main vector species in all sites, except in Nyabessang, where *An. moucheti* and *Anopheles paludis* were predominant. The mean HBR of *Anopheles* mosquitoes collected using HLC varied across the sites: 101.3 bites/human/night (b/h/n) in Simatou, 39.2 b/h/n in Gounougou, 15.9 b/h/n in Mangoum, 30.5 b/h/n in Nyabessang, and 17.5 b/h/n in Bonabéri. The HBR of *An. gambiae s.l*. was 73.9 b/h/n in Simatou, 35.6 b/h/n in Gounougou, 15.6 b/h/n in Mangoum, 3.5 b/h/n in Nyabessang, and 17.5 b/h/n in Bonabéri (Fig. 2 and Additional file 2; data 2). *Anopheles gambiae s.l*. exhibited a similar biting pattern both indoors and outdoors throughout the night in all sites with the highest biting recorded between 11:00 p.m. and 5:00 a.m. Simatou and Gounougou recorded the highest mean hourly peak biting with 8.1 bites per human per hour (b/h/h) and 4.5 b/h/h, respectively.

Trends in the monthly mean HBR of *An. gambiae s.l*. over time differed in the southern and the northern sites. In Gounougou, the lowest mean HBRs were recorded between October 2018 and January 2019 and in May and June 2019. Two peaks were observed in February (69.4 b/h/n) and August 2019 (106.0 b/h/n). In Simatou, the lowest mean HBRs were recorded from October 2018 to February 2019 and in May 2019, while the peak mean HBRs were observed in July 2019 (281.2 b/h/n) and July 2020 (327.2 b/h/n) (Fig. 2). The mean monthly HBR of *An. gambiae s.l*. was 15.6 b/h/n in Mangoum with the lowest mean HBR recorded in August 2020 (3.2 b/h/n) and the highest peak in April 2019 (42.9 b/h/n). In Nyabessang, the highest and only peak of *An. gambiae* s.l. was 14.0 b/h/n and was recorded in December 2018. From February to August 2019, the HBR of *An. gambiae* s.l. recorded was much lower (between 2.0 b/h/n and 4.8 b/h/n).

**Fig. 3:**
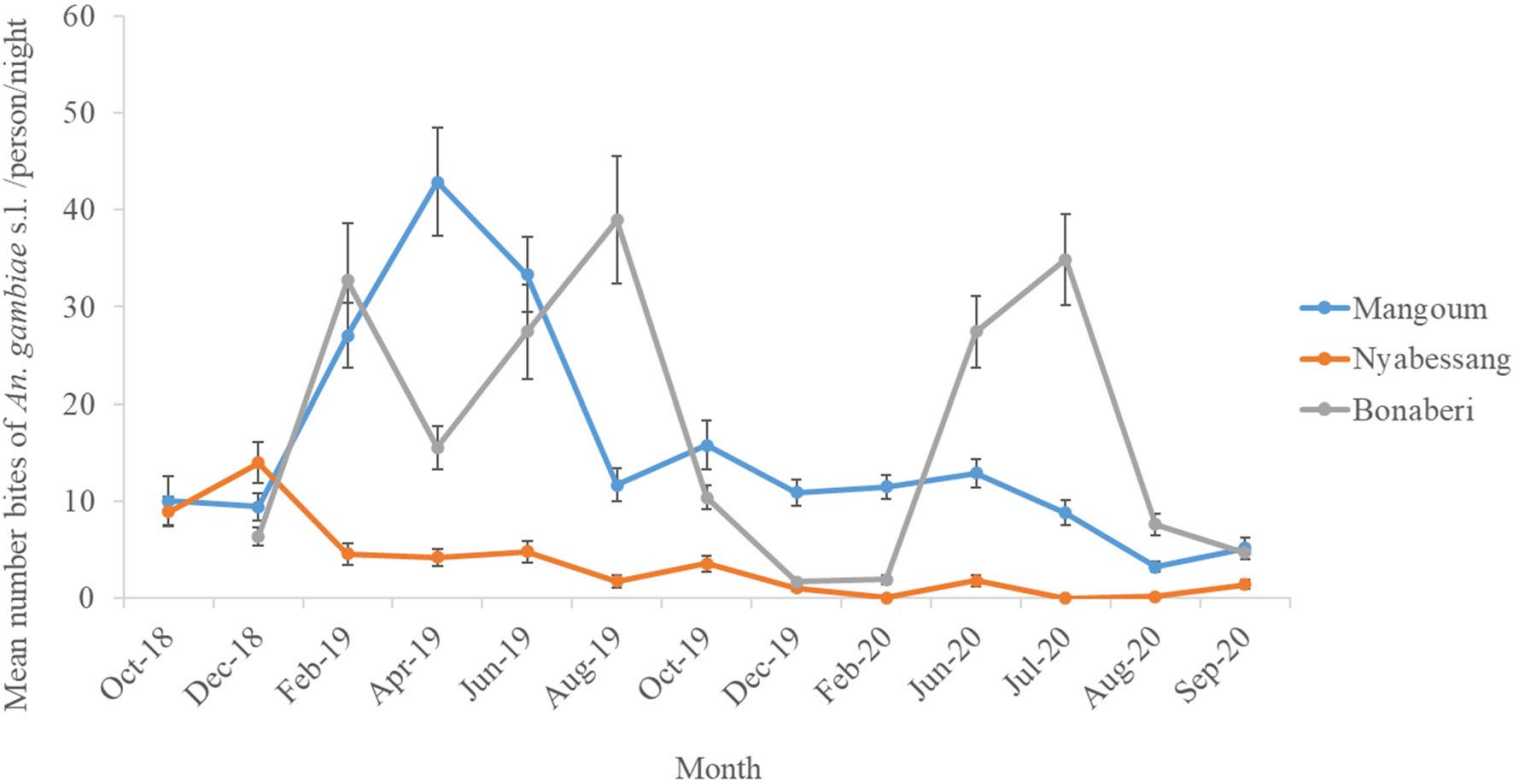
Mean monthly human biting rates of Mangoum, Nyabessang and Bonabéri (Southern sites) over the collection period (October 2018–September 2020)

Furthermore, peak biting of the predominant *An. paludis* (34.5 b/h/n) and *An. moucheti* (34.3 b/h/n) in Nyabessang was recorded in February 2019. During the second year, a peak HBR of 19.3 b/h/n was recorded for *An. moucheti* in June 2020 and 10.3 b/h/n for *An. moucheti* in August 2020, showing a replacement of vector population with *An. gambiae s.l*. which recorded its lowest density during the same period. In Bonabéri, *An. gambiae s.l*. biting peaked three times in the study period—first in February 2019 (32.7 b/h/n), then in August 2019 (39.0 b/h/n), and finally in July 2020 (35.0 b/h/n). The lowest HBR was observed in December 2019 with 1.7 b/h/n (Fig. 3 and Additional file 2: data 2).

In addition to the main malaria vector *An. gambiae s.l*., the mean HBRs of all other potential vectors were also estimated per site. In the three sites where *An. funestus s.l*. were collected, an average HBR of 0.4 b/h/n was recorded in Simatou, 2.2 b/h/n in Gounougou, and 0.1 b/h/n in Mangoum. The mean HBR of *Anopheles ziemanni* was 2.3 b/h/n in Simatou, 0.3 b/h/n in Gounougou, and 0.4 b/h/n in Mangoum. *Anopheles paludis* and *An. moucheti* were mostly found in Nyabessang with mean HBRs of 13.2 b/h/n and 10.5 b/h/n, respectively. *Anopheles nili* and *Anopheles marshallii* had mean HBRs of 2.0 b/h/n and 0.6 b/h/n, respectively. *Anopheles pharoensis* and *Anopheles demeilloni* were only collected in the northern sites, with 18.6 b/h/n and 0.5 b/h/n for *An. pharoensis* in Simatou and Gounougou, and *An. demeilloni* with a biting rate of 6.0 b/h/n in Simatou. Other potential vectors collected included *Anopheles coustani* and *Anopheles welcomei* in Simatou, and *Anopheles tenebrosus*, *Anopheles smithii*, and *Anopheles christyi* in Gounougou. (Table 3 & Additional file 1: data 1).

**Table 3:** Total mean human biting rate of *Anopheles* mosquitoes collected using human landing catches from Oct 2018 to Sept 2020 across five sites in Cameroon

Simatou			Gounougou		Mangoum		Nyabessang		Bonabéri	
Species	Total collected	Mean HBR (b/h/n)	Total collected	Mean HBR (b/h/n)	Total collected	Mean HBR (b/h/n)	Total collected	Mean HBR (b/h/n)	Total collected	Mean HBR (b/h/n)
*An. gambiae s.l.*	33,703	73.91	16,231	35.59	4859	15.57	1108	3.55	5031	17.47
*An. funestus s.l*.	186	0.41	1007	2.21	18	0.06	0	nd	0	nd
*An. ziemanni*	1041	2.28	144	0.32	110	0.35	196	0.63	1	0.00
*An. paludis*	1	0.00	0	0	2	0.01	4121	13.21	0	nd
*An. rufipes*	40	0.09	13	0.03	0	nd	0	nd	0	nd
*An. pharoensis*	8,482	18.60	248	0.54	0	nd	0	nd	0	nd
*An. coustani*	3	0.01	1	0.00	0	nd	0	nd	0	nd
*An. welcomei*	8	0.02	0	nd	0	nd	0	nd	0	nd
*An. demeilloni*	2736	6.00	0	nd	0	nd	0	nd	0	nd
*An. multicinctus*	0	nd	151	0.33	0	nd	0	nd	0	nd
*An. tenebrosus*	0	nd	9	0.02	0	nd	0	nd	0	nd
*An. smithii*	0	nd	5	0.01	0	nd	0	nd	0	nd
*An. christyi*	0	nd	1	0.00	0	nd	0	nd	0	nd
*An. moucheti*	0	nd	0	nd	0	nd	3271	10.48	0	nd
*An. marshallii*	0	nd	0	nd	0	nd	175	0.56	0	nd
*An. nili*	0	nd	0	nd	0	nd	638	2.04	0	nd
Total	46,200	101.32	17,810	39.06	4989	15.99	9509	30.48	5032	17.47

nd = not determined because the vector was not collected at those specific sites

Outdoor mean HBRs were slightly higher than indoors, but not significantly different in any sites. The mean indoor and outdoor HBRs of *An. gambiae s.l.* were 34.3 b/h/n and 36.9 b/h/n, respectively in Gounougou, 75.8 b/h/n indoors and 72.0 b/h/n outdoors in Simatou, 16.0 b/h/n indoors and 15.2 b/h/n outdoors in Mangoum, 3.4 b/h/n indoors and 3.7 b/h/n outdoors in Nyabessang, and 10.8 b/h/n indoors and 24.2 b/h/n outdoors in Bonabéri. The endophagic rate of *An. gambiae s.l*. was 50% in Simatou, 47% in Gounougou, 53% in Mangoum, and 49% in Nyabessang. Only *An. gambiae s.l*. from Bonabéri were found to bite more outdoors than indoors with an endophagic rate of 30%. The same trends were observed with *An. funestus s.l*. in Simatou (53%) and Gounougou (57%). A mean endophagic rate of about 50% was also recorded for all other *Anopheles* collected in specific sites.

### Entomological inoculation rate

A total of 15,944 *Anopheles* mosquitoes including 11,199 *An. gambiae s.l.* and 4745 other *Anopheles* were tested by ELISA, of which 496 (402 *An. gambiae s.l.* and 94 other *Anopheles*) were found with the *Plasmodium* circumsporozoite antigen, for a total average infection rate of 3.1%. Twelve *Anopheles* species were found with *Plasmodium* parasites including *An. gambiae s.l., An. funestus s.l., An. nili, An. moucheti, An. demeilloni, An. pharoensis, An. ziemanni, An. multicinctus, An. marshallii, An. tenebrosus, An. rufipes*, and *An. paludis.* The infection rates recorded across sites were as follows: Gounougou (17.0%), Simatou (13.0%), Mangoum (11.0%), Nyabessang (27.0%), and Bonabéri (4.0%). *Anopheles gambiae s.l*. from all five sites, as well as *An. ziemanni* from the four sites where it was collected, tested positive for the *Plasmodium* circumsporozoite antigen (Table 4).

**Table 4:** Entomological inoculation rate of *Anopheles* mosquitoes collected across five sites in Cameroon from October 2018 to September 2020

Sentinel Site	Species	Mean HBR	Infection Rate	Mean EIR (infected b/h/n)	Monthly Mean EIR (infected b/h/m)
**Gounougou**	*An. gambiae s.l*.	35.60	0.04	1.75	52.41
	*An. funestus s.l*.	2.20	0.03	0.07	1.98
	*An. ziemanni*	0.30	0.01	0.00	0.09
	*An. multicinctus*	0.33	0.09	0.03	0.89
	*An. tenebrosus*	0.02	0.33	0.01	0.20
**Total EIR**		**38.43**	**0.17**	**1.85**	**55.37**
**Simatou**	*An. gambiae s.l*.	73.91	0.05	2.92	87.53
	*An. funestus s.l*.	0.41	0.01	0.00	0.12
	*An. ziemanni*	2.28	0.00	0.01	0.27
	*An. demeilloni*	6.00	0.03	0.18	5.40
	*An. rufipes*	0.09	0.03	0.00	0.09
	*An. pharoensis*	18.60	0.01	0.19	5.58
**Total EIR**		**101.29**	**0.13**	**3.30**	**98.99**
**Mangoum**	*An. gambiae s.l*.	15.57	0.08	1.70	50.85
	*An. ziemanni*	0.37	0.03	0.01	0.33
**Total EIR**		**15.94**	**0.11**	**1.71**	**51.19**
**Nyabessang**	*An. gambiae s.l*.	3.55	0.05	0.21	6.32
	*An. moucheti*	10.48	0.02	0.21	6.29
	*An. nili*	2.04	0.02	0.03	1.04
	*An. ziemanni*	0.63	0.05	0.03	0.95
	*An. paludis*	13.21	0.02	0.26	7.93
	*An. marshallii*	0.56	0.11	0.06	1.85
**Total EIR**		**30.47**	**0.27**	**0.81**	**24.37**
**Bonabéri**	*An. gambiae s.l*.	17.47	0.04	0.60	18.05
**Total EIR**		**17.47**	**0.04**	**0.60**	**18.05**

EIR = entomological inoculation rate; HBR = human biting rate; b/h/n = bites/human/night; values in bold represent the total of each parameter per site

The total mean entomological inoculation rates (EIRs) varied from 18.1 infected bites/human/month (ib/h/m) in Bonabéri to 99.0 ib/h/m in Simatou. Gounougou and Mangoum recorded the second highest EIRs with 55.4 ib/h/m and 51.2 ib/h/m, respectively. Among all species, *An. gambiae s.l*. contributed the most to malaria transmission in all sites. Furthermore, the total mean EIRs of *An. gambiae s.l*. were slightly higher outdoors than indoors in Simatou (94.1 ib/h/m vs. 80.9 ib/h/m, *p = 0.381*), Mangoum (58.1 ib/h/m vs. 46.3 ib/h/m, *p = 0.771*), and Bonabéri (25.5 ib/h/m vs. 10.6 ib/h/m, *p = 0.118*), but were not significantly different at any site. Gounougou and Nyabessang recorded similar mean indoor and outdoor EIRs of 53.8 ib/h/m and 51.0 ib/h/m (*p = 0.768*), and 6.7 ib/h/m and 5.9 ib/h/m, *p = 0.408*), respectively (Additional file 2: data 2). At least two *Anopheles* species were involved in malaria transmission in four of the sites (Table 4). Simatou and Nyabessang recorded the largest number of malaria vectors with six *Anopheles* species involved in the transmission of the parasite. Gounougou recorded four vectors, while in Bonabéri, the only malaria vector found was *An. gambiae s.l*. The monthly indoor and outdoor HBRs recorded throughout the collection period, coupled with the EIRs, showed that the higher transmission period did not always coincide with the higher biting period in the southern part of the country. EIRs were highest when densities were relatively low in June 2019 in Mangoum and Bonabéri and in August 2019 in Nyabessang and all three southern sites. In contrast, EIRs in the northern sites of Simatou and Gounougou peaked when densities were highest in July and August 2019, respectively.

### Parity rate

Across all five sites, ovaries of 11,051 *Anopheles* samples were dissected during the collection period. The average parity rate across all sites was 68.9%, ranging from 57.1% (Nyabessang) to 76.4% (Gounougou) (Table 5). The mean parity rate of *An. gambiae s.l*. in Gounougou (75.1%) was significantly higher than that of the four other sites (chi^2^ = 201.3, ddl = 3, p < 0.00001). However, all *Anopheles* species dissected showed high parity rates across all sites (Additional file 3: data 3).

**Table 5:** **Parity rate of the** ***Anopheles*** **mosquitoes across five sites in Cameroon**

Sentinel site	Species	Total dissected	#Parous	% Parous
Gounougou	*An. gambiae s.l*.	2,244	1,685	75.1
	*An. funestus s.l*.	291	245	84.2
	*An. ziemanni*	50	46	92.0
	*An. pharoensis*	37	27	73.0
	*An. multicinctus*	49	38	77.5
Total		2,679	2,048	76.5
Simatou	*An. gambiae s.l.*	2,360	1,585	67.2
	*An. funestus s.l*.	89	63	70.8
	*An. ziemanni*	278	108	38.9
	*An. rufipes*	15	11	73.3
	*An. pharoensis*	1,469	1,090	74.2
	*An. welcomei*	13	10	76.9
	*An. demeilloni*	393	281	71.5
Total		4,619	3,150	68.2
Mangoum	*An. gambiae s.l*.	650	436	67.1
	*An. ziemanni*	13	9	69.2
	**Total**	665	445	66.9
Nyabessang	*An. gambiae s.l.*	369	228	61.8
	*An. ziemanni*	59	38	64.4
	*An. paludis*	527	270	51.2
	*An. moucheti*	664	381	57.4
	*An. marshallii*	27	15	55.6
	*An. nili*	137	86	62.8
Total		1,785	1,019	57.1
Bonabéri	*An. gambiae s.l*.	1,301	944	72.6
Total		1,303	945	72.6

### Malaria vector resting behaviour

Eleven *Anopheles* species were collected resting indoors using PSCs representing 18.6% (25,940) of the total *Anopheles* mosquitoes collected at all sites during the collection period. Similar to HLCs, Simatou and Gounougou recorded a more diverse and higher number of different *Anopheles* species collected through PSCs compared to other sites. Seven of the 11 species collected overall were found in the two northern sites and included *An. gambiae s.l*., *An. funestus s.l.*, *An. ziemanni*, *An. rufipes*, *An. pharoensis*, *An. hancocki*, and *An. demeilloni.* Two *An. multicinctus* were also collected in Gounougou while *An. moucheti* and *An. nili* were found in Nyabessang. In Mangoum and Bonabéri, *An. gambiae s.l*. was the only species collected.

### Indoor resting density across sites

The average density per room of *Anopheles* mosquitoes resting indoors (IRD) was 17.1 females/room (f/r) (25,940 total females/1520 rooms visited). Table 6 describes the IRD per site. The highest mean IRD was recorded in Simatou (39.6 f/r) in the north, while the lowest was observed in Bonabéri (0.04 f/r) in the south and varied by month and season. The mean IRD of *An. gambiae s.l*. was 34.7 f/r in Simatou and 23.4 f/r in Gounougou. The highest was observed in July 2020 (135.2 f/r) in Simatou and in July 2019 (77.5 f/r) in Gounougou. In the southern sites, the mean IRDs were low compared to the northern sites with a mean of 1.8 f/r in Mangoum, 0.6 f/r in Nyabessang, and 0.04 f/r in Bonabéri. Seasonal variation was also observed in the southern sites where the highest IRD was recorded in October 2018 (4.3 f/r) in Mangoum, in July 2020 (0.2 f/r) in Bonabéri, and in September 2020 (1.2 f/r) in Nyabessang (Additional file 3: data 3).

**Table 6:** Mean indoor resting density of *Anopheles* mosquitoes collected by pyrethrum spray catches across five sites from October 2018 to September 2020

	Simatou		Gounougou		Mangoum		Nyabessang		Bonabéri	
Species	Total collected	Mean IRD (f/r)	Total collected	Mean IRD (f/r)	Total collected	Mean IRD (f/r)	Total collected	Mean IRD (f/r)	Total collected	Mean IRD (f/r)
*An. gambiae s.l.*	13,190	34.7	8905	23.4	467	1.8	140	0.6	9	0.04
*An. funestus s.l.*	112	0.3	1127	3.0	0	nd	10	0.04	0	nd
*An. rufipes*	695	1.8	213	0.6	0	nd	0	0	0	nd
*An. ziemanni*	13	0.03	5	0.01	0	nd	5	0.02	0	nd
*An. pharoensis*	82	0.2	0	nd	0	nd	0	0	0	nd
*An. multicinctus*	0	nd	2	0.01	0	nd	0	0	0	nd
*An. pharoensis*	0	nd	3	0.01	0	nd	0	0	0	nd
*An. hancocki*	11	0.03	0	nd	0	nd	0	0	0	nd
*An. moucheti*	0	0	0	0	0	0	10	0.03	0	0
*An. nili*	0	0	0	0	0	0	1	0.00	0	0
*An. demeilloni*	940	2.5	0	nd	0	nd	0	0	0	nd
Total	15,043	39.6	10,255	27	467	1.8	166	0.6	9	0.04

IRD = indoor resting density; nd = not determined because the vector was not collected at those specific sites

### Host preference of *Anopheles* species across sites

Nine of the 18 *Anopheles* species collected from the five sites were screened for blood meal sources to detect if the bloodmeal taken was from a human, cow, sheep, chicken, pig, or horse. A total of 2994 blood-fed *Anopheles* mosquitoes were analyzed using ELISA, including 2144 *An. gambiae s.l*., 225 *An. funestus s.l*., 252 *An. rufipes*, 83 *An. demeilloni*, 31 *An. pharoensis*, 246 *An. ziemanni*, 2 *An. moucheti*, 1 *An. nili*, and 10 *An. hancocki.* Only 1,151 of the blood-fed mosquitoes analyzed were found to have fed on humans, giving a HBI of 38.4%. The overall HBI varied from 34.3% in Gounougou to 82.3% in Mangoum. The HBIs of *An. gambiae s.l*. in Mangoum (82.3%) and Nyabessang (64.2%) located in the south were significantly higher than those in the two northern sites (chi^2^ = 14.18, ddl = 3, p < 0.000001). For *An. funestus s.l.*, the HBI was 44.4% among the samples collected in Simatou and Gounougou. Out of the 252 *An. rufipes* tested in Simatou and Gounougou, only 12 (4.8%) were found with human blood meal while *An. pharoensis* tested in Simatou showed a HBI of 40% (12/30).

### Vectorial capacity

The vectorial capacity, described as the ability to serve as a vector, was determined for seven species (*An. gambiae s.l*., *An. funestus s.l.*, *An. ziemanni*, *An. rufipes*, *An. pharoensis*, *An. demeilloni*, and *An. welcomei*) in Simatou, six in Gounougou (*An. gambiae s.l*., *An. funestus s.l.*, *An. ziemanni*, *An. rufipes*, *An. pharoensis*, and *An. multicinctus)*, five in Nyabessang (*An. gambiae* s.l., *An. ziemanni*, *An. marshallii, An. moucheti*, and *An. nili)* and only *An. gambiae* s.l. in Mangoum and Bonabéri. *Anopheles gambiae s.l*. showed the highest vectorial capacity in all sites except Nyabessang, where *An. moucheti* represented the main potential malaria vector with an index of vectorial capacity of 2.49 versus 0.54). Vectorial capacity in Simatou (32.0) and Gounougou (19.02) indicate a higher capacity of *An. gambiae s.l*. to transmit malaria compared to the other *Anopheles* species reported with sporozoite infections. In contrast, *An. gambiae s.l.* was the main vector collected in Mangoum (9.81) and Bonabéri (10.12) with similar index on vectorial capacity (Table 7). *Anopheles funestus s.l*. was the second highest contributor of persistent malaria in Gounougou, while *An. pharoensis* and *An. demeilloni* represented the two secondary vectors in Simatou (Additional file 4: data 4).

**Table 7:** Vectorial capacity of *Anopheles* species collected across five sites in Cameroon

	Simatou	Gounougou	Nyabessang	Bonabéri	Mangoum
*An. gambiae s.l.*	32.001	19.017	0.542	10.139	9.811
*An. ziemanni*	0.009	0.011	0.000	nd/nc	nd/nc
*An. funestus s.l.*	0.401	5.359	nd/nc	nd/nc	nd/nc
*An. pharoensis*	8.115	0.000	nd/nc	nd/nc	nd/nc
*An. rufipes*	0.012	0.307	nd/nc	nd/nc	nd/nc
*An. welcomei*	0.000	nd/nc	nd/nc	nd/nc	nd/nc
*An. demeilloni*	5.145	nd/nc	nd/nc	nd/nc	nd/nc
*An. multicinctus*	nd/nc	0.000	nd/nc	nd/nc	nd/nc
*An. moucheti*	nd/nc	nd/nc	2.491	nd/nc	nd/nc
*An. marshallii*	nd/nc	nd/nc	0.000	nd/nc	nd/nc
*An. nili*	nd/nc	nd/nc	0.000	nd/nc	nd/nc

nd/nc = not determined because either the vector was not collected at those specific sites, or the mosquitoes collected did not undergo ovary dissection

## Discussion

Entomological vector surveillance is key to describing vector populations and behaviour and thereby informing the development of appropriate vector control strategies and tailored deployment of tools. This study, conducted in different ecological and geographical areas of Cameroon, indicated a high diversity and density of *Anopheles* species in the country. The longitudinal vector monitoring conducted over about two consecutive years provides concrete density, diversity, and transmission trends of the different malaria vectors across the country. Of the 21 *Anopheles* species and sub-species collected, 12 species were found to be positive for *P. falciparum* sporozoites. Other recent studies conducted in Cameroon have revealed a high diversity of malaria vectors distributed across different geographical locations within the country [12, 17-19]. However, *An. gambiae s.l*. was the dominant vector and was found in all sites. *Anopheles moucheti* and *An. nili* were observed only in Nyabessang, which is surrounded by large rivers and dams offering suitable breeding sites for the development of larvae of these two species. Among the species of the *An. gambiae* complex, *An. arabiensis* was found in the two northern sites where the climate is drier than in the southern regions, which corresponds to reports from other sub-Saharan African countries where *An. arabiensis* was also found in drier areas [34, 35]. Furthermore, *An. gambiae s.s.* was predominant in Mangoum and Nyabessang while *An. coluzzii* represented the main species of the complex found in the other three sites. It is known that *An. gambiae s.s*. prefer larval habits with lots of sun exposure, while *An. coluzzii* are typically found in the man-made areas such as rice fields and more humid areas [36, 37]. The findings of this study corroborate with previously reported data [12, 18, 19], as Simatou and Gounougou are rice cultivation areas and Bonabéri is in the southern humid area. Similar results have also been reported from previous studies conducted in comparable eco-geographical areas in the country [38], though this study assessed vector bionomics over two consecutive years. Reviewing the trends over this period can help decision makers to assess and determine not only the tools that will be most effective, but also the optimal timing of deployment to achieve the desired impact. For example, the eco-geographical location of Nyabessang, surrounded by many rivers and dams with a high rainfall, favored the development of *An. paludis* and *An. moucheti* over leading *An. gambiae s.l*. at a specific period of the year. The high density of both species observed during the same period of the year could indicate the need for integrated strategies to control all species. On the other hand, Mangoum recorded the lowest species diversity with predominantly *An. gambiae s.l*. And few *An. ziemanni* recorded throughout both years of collections. This could be due to the location of the site and farming activities including corn and tomato gardening. Mangoum is a humid and sunny area located within a forest savannah, which is favourable for *An. gambiae s.l*. breeding sites, implying that vector control tools that target *An. gambiae s.l*. will likely be effective, such as any appropriate combination of ITNs.

The HBR of the *Anopheles* species varied seasonally at each site, which could be related to the eco-geographical location of the sites and peaked with either increasing rainfall and/or rice cultivation. Rainfall and rice paddies are known factors contributing to an increase in biting and consequently an increase in the malaria incidences in endemic countries [39, 40]. Biting in both Gounougou and Simatou peaked during the rainy season, which coincides with rice cultivation. This trend was observed over both survey years and highlights the need for malaria control strategy implementation, particularly during the peak transmission period which seems to recur from year to year. The NMCP in Cameroon has initiated seasonal malaria chemoprevention (SMC) in the northern regions [41] and provides free malaria treatment of children under five across the country. In addition, ITNs are distributed through mass campaigns and routine channels. However, it may be necessary to consider additional vector control measures such as complementary larval source management (LSM) or indoor residual spraying (IRS) where feasible. An impact evaluation may help determine if LSM could help control the diversity of *Anopheles* in areas where rice cultivation is conducted, such as in Simatou and Gounougou. In contrast to the northern sites, two biting peaks were recorded at the sites in southern part of the country, where two rainy seasons are observed yearly. Interestingly, all vectors showed slight (but not significantly different than indoor) outdoor biting patterns over both years and at all sites, even though several animal shelters were found in the northern sites, which could contribute to outdoor feeding of the vectors [42, 43]. Furthermore, the endophagic rates recorded in Gounougou and Simatou were lower than those of the southern sites because of the presence of a substantial number of cattle farms. However, HBRs were still high in Cameroon compared to some sub-Saharan African countries with similar geographical and climatic conditions [44, 45, 46, 47, 48].

Though malaria transmitted by *An. gambiae s.l*. was similar indoors and outdoors at all sites, the highest transmission was observed in Simatou, where six *Anopheles* species were found infected and a higher HBR was recorded compared to the other sites. This multiplicity of vectors could continue to worsen given recent reports on the potential transmission by sub-species of *An. funestus s.l*. in addition to those known in the *An. gambiae* complex [49, 50]. Though the diversity of malaria vectors has been described in the country [12, 17, 51], no specific vector control measures targeting the various species have been developed to date. All control efforts are channeled towards the main vector *An. gambiae s.l*., with the expectation that they will also have effects on the other vectors. However, the vectorial capacity of other vectors that are currently considered as secondary vectors needs to be closely monitored. Furthermore, this diversity of *Anopheles* vectors constitutes a cause for concern, considering that the current vector control interventions only target mostly endophagic and endophilic *An. gambiae s.l*. This strategy could alter vector dynamics, creating opportunities for niche partitioning and for other vectors to fill in the gap left by reduced populations of *An. gambiae s.l.* As observed in Nyabessang, *An. moucheti* and *An. paludis* yielded higher vectorial capacity and entomological inoculation rates compared to *An. gambiae s.l*. This may require deeper investigation into the ecology, transmission, and epidemiological impact of these vectors for targeted vector control interventions. Despite climate difference between the two northern sites, the pattern of the malaria transmission was similar, and all vectors observed had high parity rates suggesting they live long enough to transmit the disease, as the parity rates recorded at all sites were high for most of the vectors. This observation suggests that the current vector control tools implemented by the NMCP may have limited impact on the vectors. Cameroon recommended universal coverage and mass distribution of pyrethroid-only ITNs for a decade before introducing new types of nets during the 2022 mass ITN distribution campaigns. Even though some positive results were recorded on the decrease of morbidity due to malaria, more efforts are needed to reach elimination of the disease in the country. The use of ITNs was reported to be low among the target populations of Cameroon [52], indicating the need for social and behaviour communication programs to be undertaken by the NMCP.

## Conclusion

Cameroon has a diverse and high density of *Anopheles* species, with *An. gambiae s.l*. as the main malaria vector in most geographical regions of the country. However, in this survey, *An. moucheti* and *An*. *paludis* were the main malaria vectors in Nyabessang. Seasonal variations of HBRs and the indoor resting density of *An. gambiae s.l*. were observed in all sites. *Anopheles gambiae s.l*. was observed to bite more indoors in Mangoum and more outdoors in Gounougou, Bonabéri, and Nyabessang, with biting occurring until the early morning hours at all sites.

Eleven *Anopheles* species and subspecies were involved in malaria transmission and *An. gambiae s.l*. highly contributed at all sites, except in Nyabessang, where *An. moucheti* and *An. paludis* accounted for 25.9% and 32.1% of EIR, respectively. This study highlights the urgent need for integrated vector control interventions considering all potential vectors to reduce malaria transmission and burden in Cameroon. Based on these results, a literature review, and the insecticide resistance monitoring data recorded across the country, the NMCP conducted the 2022 ITN mass distribution using PBO ITNs and dual active ingredient Interceptor G2 ITNs in regions with high malaria endemicity, high transmission, and high insecticide resistance intensity. The data could also support the deployment of IRS in targeted sites with timing determined by the trends observed over the collection years. Targeted LSM could be an additional option to reduce the peak biting and transmission in northern areas, where rice cultivation increases the mosquito population density, but should be implemented in the context of an impact evaluation.

## Supplementary Information

**Additional file 1:** Total *Anopheles* mosquitoes collected and human biting rates per site.

**Additional file 2:** Mean indoor, outdoor, and total entomological inoculation rate per site.

**Additional file 3:** Mean indoor resting density of *An. gambiae s.l*. per site.

**Additional file 4:** Parity and vectorial capacity estimations.

## Data Availability

The datasets used and/or analysed during the current study are available in the supplementary data set and could also be provided by the corresponding author on reasonable request.
